# Gender euphoria reimagined: toward an Extended Theory of Trans-Identity

**DOI:** 10.3389/fpsyg.2026.1738195

**Published:** 2026-03-13

**Authors:** Mary Angelene J., Alamelu C.

**Affiliations:** School of Social Sciences and Languages, Vellore Institute of Technology, Chennai, India

**Keywords:** artistic expression, gender euphoria, integration, theory extension, transgender identities

## Abstract

**Introduction:**

The paper focuses on developing an Extended Theory of Trans-Identity integrating Nagoshi et al.'s trans-identity theory with the creative expression of the self to explore gender-euphoric experiences.

**Method:**

Having explored Austin et al.'s research on the artistic expression of trans people, the article attempts to explore the creative manifestation of identity as an important aspect of trans-identity formation, alongside Nagoshi et al.'s trans-identity theory. The synthesis of four aspects, including physical embodiment, self-construction, social construction, and the creative manifestation of the identified gender leading to the attainment of gender-euphoric factors identified by Austin et al. and Leitch et al., culminates in an Extended Theory of Trans-Identity. The theoretical framework is applied to *I Am Vidya: A Transgender's Journey* (2013), the autobiography of Living Smile Vidya, who is an eminent trans theatre artist and activist from Tamil Nadu, India. Furthermore, the use of a literary text to validate the Extended Theory draws on Schilling's concept of theory extrapolation from literature. The exemplifying textual analysis of the dynamic role of the four aspects of trans-identity in asserting transness and achieving gender euphoria attempts to substantiate the proposed theoretical extension.

**Results:**

The results indicate that identity construction through the creative aspect, in combination with the biopsychosocial aspects, contributes to the utmost attainment of gender euphoria.

**Discussion:**

Therefore, the article formulates an extended version of the trans-identity framework, emphasising the inclusion of creativity in the gender-affirmative journeys of trans people.

## 1 Introduction

Transgender people undergo enormous struggles and face complex situations in transphobic spaces while expressing gender identities that are internally recognized (Lewis et al., 2023). Existing research delineates transgender identities through essentialist, psychoanalytic, transnormative, social constructivist, postmodernist, posthumanist, feminist, queer, and ecological approaches. Recently, narrative approaches have gained prominence in the field of trans studies as a means of interpreting trans-identity formation from various perspectives (Doyle, 2022; McAdams, 2011). To comprehend the formation, expression, and affirmation of identities, trans studies seek to transcend “the boundaries constructed by their culture to define and contain that gender” (Stryker, 2017, p. 1). On this note, Nagoshi et al.'s (2013) trans-identity theoretical framework, which is informed by and extends beyond feminist and queer scholarship, includes bodily experiences, psychological adaptations, and socially adopted behaviors that are unique to transgender individuals. This framework has been employed by scholars in social work, media, and other domains to raise awareness, highlight the living conditions of trans communities, and emphasize the significance of media and literary representations of trans experiences.

The extended version blends the trans-identity theoretical framework with the creative construction of identity, which, in turn, leads to gender euphoria. Gender euphoria, within the trans community, is a term that describes the positive experiences and emotions unique to trans individuals (Austin et al., 2022b). A trans individual experiences positivity when expressing the identified self without inhibition, and art provides a space to creatively express one's authentic self and gain social recognition and acceptance (Quach and Kwon, 2025). Therefore, the theoretical development article seeks to formulate a framework that integrates the existing model of physical, psychological, and social construction of the self with the creative construction of the self to facilitate gender euphoria, drawing on literary-based substantiation. These euphoric moments play a key role in affirming the sense of self, as “coming to terms with who you are can be more than avoiding someone you don't want to be” (Dale, 2021, p. 4).

## 2 Methods

The theoretical construction adopts a methodology comprising the following steps:

- Step 1: Expand the theory of trans-identity proposed by (Nagoshi et al. 2013) by incorporating Austin et al.'s (2022a) insights on the artistic expression of the self in trans individuals.
- Step 2: Synthesize the four aspects—physical, psychological, social, and creatively constructed—of the self, as derived from the research of (Nagoshi et al. 2013) and (Austin et al. 2022a).
- Step 3: Integrate these aspects with the four factors contributing to gender euphoria identified from Austin et al.'s (2022b) and Leitch et al.'s (2025) research, culminating in an Extended Theory of Trans-Identity.
- Step 4: Corroborate the validity of the framework by employing textual analysis to closely examine the contents of the autobiography, *I Am Vidya: A Transgender's Journey* (2013), which recounts the first-hand experiences of a trans woman.

### 2.1 Extended Theory of Trans-Identity

The theoretical framework of trans-identity, proposed by (Nagoshi et al. 2013) in the book *Gender and Sexual Identity: Transcending Feminist and Queer Theory*, is an attempt to advance the relational model proposed by (Shotwell and Sangrey 2009). The trans-identity theory transcends the demarcations of feminist and queer studies (Billard and Zhang, 2022) and posits that transgender identities can be understood through the narratives of lived experiences (Monro, 2000; Nagoshi et al., 2023), which can be grouped into three overlapping aspects of gender identity: physical embodiment, self-constructed identity, and socially constructed identity. These three aspects of identity generate distinctive experiences and agency (Nagoshi et al., 2013). In addition, art also emerges as a valuable medium for constructing authentic identities and facilitating the agency needed to affirm one's true self (Austin et al., 2022a). Hence, the theoretical framework used in this paper integrates all these aspects for trans-identity construction.

#### 2.1.1 Physical embodiment

The first aspect, physical embodiment, can be understood through the lens of (Stryker 2022), who argues that “bodies are rendered meaningful only through some culturally and historically specific mode of grasping their physicality that transforms the flesh into a useful artifact” (p. 76). Her contemporary, (Braidotti 1994), also affirms that the body serves as a corporeal medium for expressing gender. The “corporeal signification” phenomenon leads to the acquisition of social experiences associated with identified genders (Butler, 1999, p. 139; Hines, 2007; Muthukrishnan and Sunitha, 2025; Salamon, 2010). Originating from the essentialist principles of gender, (Nagoshi et al. 2013) argue that gender is determined by society based on an individual's primary and secondary sexual characteristics. Male-to-female trans individuals may undergo sex reassignment procedures such as hormone therapies to block testosterone and induce estrogen, breast augmentation, and vaginoplasty, commonly known as top and bottom surgeries (Kuper et al., 2018; Murad et al., 2010; Nassar et al., 2025). Conversely, female-to-male trans individuals may undergo hysterectomy, breast reduction surgeries, and other procedures (Nassar et al., 2025; Rachlin et al., 2010). Therefore, trans embodiment is a reflection of profoundly restructured lives that emerge from reshaped and reorganized bodies (Nagoshi et al., 2013, 2023; Schrock et al., 2005).

#### 2.1.2 Self-constructed identity

(Kuper et al. 2018) argue, based on their qualitative findings, that transition is the quest to have what suits one or fits one, working toward being how one wants to be and embracing the true sense of one's gender identity. It results in a self-constructed identity that emerges from resistance to dichotomy and subverts socially imposed identities (Hines and Sanger, 2010; Muthukrishnan and Sunitha, 2025). The exaggerated behavior displayed while expressing a self-constructed gender exemplifies the manifestation of agency against the assigned gender by conforming to the social roles of the identified gender (Nagoshi et al., 2013). Trans narratives of self-constructed identity are characterized by the rejection of assigned gender labels, the assertion of a self-constructed gender, the insistence that others acknowledge it, and the advocacy for related rights (Alessi et al., 2021). The “transcendent stories” of trans people destabilize rigid gender binaries by rejecting previous experiences of the assigned gender and redefining the self, body, and social narratives aligning with the identified gender (Ekins and King, 2006, p. 181; Gottzén and Straube, 2016; Heckler and Nickels, 2025).

#### 2.1.3 Socially constructed identity

The aim of trans-identity affirmation is not only to destabilize the gender system but also to make it fluid and inclusive, as one's identity is not only concerned with self-identification but also inevitably intertwined with various power structures and social institutions that influence one's belonging to a particular social group (Nagoshi et al., 2013; Shields, 2008). Non-conformity to social norms of identity creates “dissidence” and leads to gender bias, discrimination, and oppression. According to Nagoshi et al.'s (2013) theorization, the sociocultural environment of a person essentializes an objective identity defined by social norms and expectations pertaining to the categorized genders and enforces conformity. Trans people, to attain a sense of belonging to the identified gender, adopt, and adhere to mutable characteristics, such as dressing, makeup, interests, activities, and traits associated with gender stereotypes, recognizing the social interpretation attached to these practices (Kuper et al., 2018; Ogle et al., 2023). These gender-expressive “acts, gestures, desire, and performative attributes” facilitate the “corporeal signification” (Butler, 1999, p. 139) of an identified gender, creating a gendered social identity through repeated performances that are consistent with the cultural perceptions of the identified gender (Nagoshi et al., 2013, 2023). However, the essentialised identity adopted should not be reductively viewed as upholding gender binaries but as a reclamation of one's individual identity on one's own terms—a distinctly transgender experience (Baldino, 2023).

#### 2.1.4 Creatively constructed identity

The article, adding to the three aspects of trans identity formation: physical embodiment, self-constructed identity, and socially constructed identity, introduces creatively constructed identity as the fourth aspect. It attempts to show how spaces of creative expression encourage a trans person to explore the sense of self, gender, and sexuality, ultimately supporting the emergence of true gender identity (Pelton-Sweet and Sherry, 2008; Souza, 2023). Similar to other aspects, creative expression manifests the becoming of bodies, thoughts, feelings, and experiences (Austin et al., 2022a). Moreover, indulging in creative practices empowers trans people to regulate emotions, fostering the development of self-esteem and social skills (Chevalier-Amy, 2020; Lyons et al., 2018; MacLeod et al., 2016; Rhodes and Schechter, 2014), which, in turn, promotes self-acceptance, comfort in one's body, and social recognition (Fang et al., 2024; Rooke, 2010). It also helps trans people connect with peers and others as it provides the agency and space to narrate trans experiences, affirm identities, and challenge misconceptions (Austin et al., 2022a). As art can inspire viewers to think, reflect, empathize, and change perceptions, it can engage people in recognizing the struggles of marginalized identities (Mihai, 2018; Sari, 2025). Therefore, creative narratives of identity also have the power to resist prevailing societal narratives, as counter-narratives of identities, survival, and history are created through art (Austin et al., 2022a; Fobear, 2017; Stornaiuolo and Thomas, 2018). However, it is important to note that trans individuals can choose to disengage from these practices if they do not contribute to a positive sense of self (Parker, 2024).

#### 2.1.5 Integration of the four aspects of trans-identity and factors of gender euphoria

This article attempts to theorize the convergence of the four aspects of trans-identity to promote gender euphoria in trans people. This synthesis can be validated as an extension of trans-identity theory, based on the arguments articulated in the chapter “Theory Extrapolated from Literature” in Erik Schilling's *Theorizing Literature: Literary Theory in Contemporary Novels - and Their Analysis* (2024). Schilling identifies two primary forms of theory extrapolation from literature: metaization and the creation of a theory. This theory formation employs the latter to extend the already established trans-identity theory (Nagoshi et al., 2013). Although it can be developed from non-literary sources, the framework chooses an autobiography, a literary text, due to its accessible and vivid representation of identity formation and gender euphoria attainment in transgender individuals. Autobiography may be treated as a resource of knowledge, where the practices of “doing gender” can be discovered in personal and unique stories of life (Järviluoma et al., 2003, p. 63), and is vital for “undoing internalized oppression” (Sayre, 2022, p. 3). Literary texts, as pointed out by (Schilling 2024, p. 195), are not only “an object” to be applied to and scrutinized by theoretical constructs but also “a generative site” for developing new theories or for modifying and extending existing ones.

**Figure 1:**
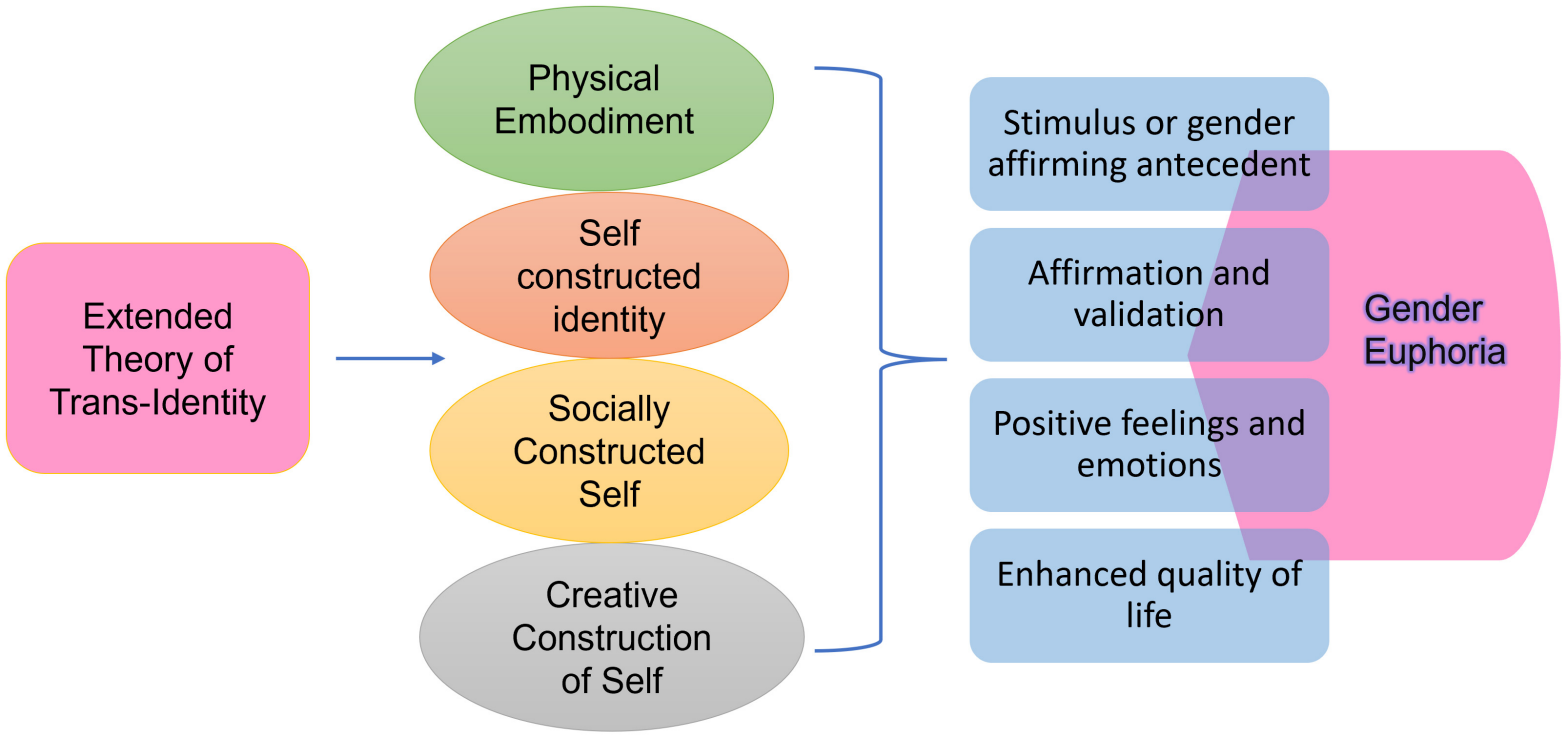
Extended Theory of Trans-Identity: Integration of the four facets of trans-identity leading to the four factors of gender euphoria.

The Extended Theory of Trans-Identity incorporates gender euphoria into the formation of trans identities. Previous research defines gender euphoria as the joyful feeling of contentment and elation that arises when an individual's gender identity, gender expression, and affirmation are authentic and congruent with the inner gendered self (Ashley and Ells, 2018; Beischel et al., 2022; Bradford et al., 2021). The necessary conditions for trans individuals to experience gender euphoria are drawn collectively from Austin et al.'s (2022b) grounded theory research and Leitch et al.'s (2025) qualitative research on gender euphoria, forming the basis for extending the theory. The occurrences of gender euphoria in trans individuals are linked to the following conditions: gender euphoria occurs (1) due to a stimulus or gender-affirming antecedent, which refers to any encounter or incidents that precede positive thoughts or reactions about one's own gender identity; (2) as a response to affirmation and validation, either through self-affirmation of one's own becoming or validation received externally; (3) when intense positive feelings and emotions are experienced as a result of affirming experiences and accomplishing personal milestones in constructing identity; and (4) when experiencing an enhanced quality of life that was unavailable or inaccessible prior to transitioning into the identified gender (Austin et al., 2022a,b; Leitch et al., 2025). Gender euphoria can be attained when trans identities are manifested through the integration of physical embodiment, self-constructed identity, socially constructed self, and creatively constructed self, as shown in Figure 1.

### 2.2 Significance of the Extended Theory of Trans-Identity

In addition to the biopsychosocial realignments of trans people, artistic exploration can also play a vital role in the transition journey by providing an avenue for self-expression and eliciting empathy and acceptance. As (Szasz 1973) notes, “The self is not something that one finds. It is something that one creates” (p. 49). Therefore, spaces of creative expression, where a trans individual can experience positive reinforcement of one's own essence, can have a significant impact on the development of one's gender identity (Austin et al., 2022a). Artistic manifestation of the perceived self can provide the strength to express a self-asserted gender in social spaces. Thus, engaging in creative practices alongside preexisting transition-related practices can lead to euphoric experiences of exhibiting authentic gender identities.

## 3 Applying the Extended Theory of Trans-Identity to the autobiography, *I Am Vidya: A Transgender's Journey* (2013)

The Extended Theory of Trans-Identity is applied to an autobiography, as it is one of the most salient “vehicles for exploring the human realm in all of its depth, complexity, and richness” (Given, 2008, p. 74). The selected autobiography, *I Am Vidya: A Transgender's Journey* (2013), is Vidya's account of her experiences as a trans woman from India. She is a prominent theater artist and actress, has worked as an assistant director in Tamil and Malayalam films, and, more importantly, is a trans and Dalit rights activist. The autobiography has been originally written in Tamil and later translated into seven other languages; it has been also adapted into the Kannada film *Naanu Avanalla...Avalu* (2015). This significant literary piece is closely analyzed to identify moments of gender euphoria, corresponding the four aspects of identity, through the lens of the Extended Theory of Trans-Identity.

### 3.1 Physical embodiment

Constructing one's body in accordance with the subjective sense of gender identity constitutes physical embodiment. (Merleau-Ponty 2013) notes that the physical embodiment of gender identity is a social practice that generates experiences that are socially, culturally, and historically specific (Baldino, 2023). When Vidya, who was assigned male at birth, embodies femininity, she is often perceived as unnatural. As stated by (Butler 2004), society rigidly adheres to a “matrix of intelligibility” that molds the binary structures of sex, gender, sexuality, and social roles, rendering any deviation unintelligible (Muthukrishnan and Sunitha, 2025; Nair and Francis, 2024). The autobiographical account of Vidya provides instances where Vidya chooses to combat this unintelligibility through physical embodiment, generating euphoric factors such as a stimulus or gender-affirming antecedent and positive emotions.

#### 3.1.1 Stimulus or gender-affirming antecedent

Trans women in India practice Nirvana, a procedure involving the removal of male genitalia (Mishra, 2023), to eliminate the incongruence between the desired social roles and the physical attributes.

“What we undergo here is merely castration under local anaesthesia—and that too without government approval. An illegal procedure, its fruits include a lack of social approval, such as denial of jobs and opportunities for higher education. Transgenders in India have no option but to resort to begging or prostitution. Why then should we undergo this illegal operation? One would be right to ask such a question... We are women at heart desperately seeking to delete or erase our male identity. That is why we crave the surgical procedure that will give us the bodily likeness of that female identity” (Vidya, 2013, p. 89).

Here, Vidya combats dysphoria and attains a sense of gender congruence through her physical transition. She writes, “They had removed that part of me over which I had shed silent tears of rejection from as far back as I could remember—my penis and my testicles had been excised” (Vidya, 2013, p. 12). When there is internal alignment, she experiences gender euphoria (Kai and Devor, 2022), which she exclaims as, “Ah! Nirvana! The ultimate peace!” (Vidya, 2013, p. 12).

#### 3.1.2 Intense positive feelings and emotions

When one feels content and connected to the physical body, one embodies joy and a positive sense of self (Kulesza et al., 2025).

“Inside, I was at peace. It was a huge relief. I was now a woman: mine was a woman's body. Its shape would be what my heart wanted, had yearned for. This pain would obliterate all earlier pains” (Vidya, 2013, p. 13).

Vidya describes her physical transition as a step toward acknowledging her womanhood. According to (Paz Galupo et al. 2021b), gender-affirming procedures significantly decrease bodily discomfort among trans people.

### 3.2 Self-constructed identity

The self-constructed gender identity accommodates, as (Serano 2016) posits, both “what we consciously choose to identify as, and subconsciously feel ourselves to be” (p. 78). Inner thoughts and dialogues emerging from experiences influence behavior (Baldino, 2023; Beck and Beck, 2011). Here, Vidya's transition from an obsession with becoming a woman to being a woman is traced through the process of self-construction of identity. This self-constructed identity gives rise to a stimulus or gender-affirming antecedent, affirmation and validation, and intense positive emotions.

#### 3.2.1 Stimulus or gender-affirming antecedent

Trans people imbibe the stereotypical roles of different genders from fellow members of society and media (Mishra, 2023). These roles serve as reference points for building desirable gender identities.

“My boyhood obsession with film heroines and my secret pleasure in cross-dressing to look like them intensified at this point rather than being reduced. I was seventeen—no amount of teasing had any ability to make me behave differently by then” (Vidya, 2013, p. 27).

Throughout the autobiography, Vidya can be observed building her fantasy world by taking inspiration and cues from films for the social behaviors associated with being a woman.

“Once, I had lost myself before the mirror in admiration after the make-up artist had put lipstick on me... The mirror may reflect your outer appearance to you and others like you, but in the case of Tirunangais (term used in Tamil for trans woman) it portrays their innermost feelings and turbulence, their essential femininity, displaying all... I was self-forgetfully drinking in my own beauty as reflected by the mirror, encouraged by my solitude” (Vidya, 2013, p. 49).

The above instance suggests that “sensory observation of the body” (Kondelin, 2017, p. 26; Ogle et al., 2023) can act as a stimulus for gender euphoria. Vidya is seen to be preening before the mirror and admiring herself, knowing fully well that she is born to live as a woman.

#### 3.2.2 Affirmation and validation

Presenting oneself in alignment with the social expectations of one's identified gender affirms one's innate self while subverting socially imposed identities (Baldino, 2023).

“Gradually, both family and neighbours started noticing my unusual behaviour. My habits of wearing drag, bathing with the towel wrapped around my chest, tying a towel around my head to dry my imaginary long hair—all this was now public knowledge. My voice was still soft and effeminate, and I tended to blush, gesture and walk like a woman, too” (Vidya, 2013, p. 24).

The act of donning the attire of a woman and presenting herself to society as a woman subverts the socially imposed male identity. Vidya's self-affirmation as a woman is perceived as undesirable because it threatens the symbolic social and cultural meanings attached to male or female bodies (Muthukrishnan and Sunitha, 2025), as “it is not improbable that a male-dominated society cannot tolerate a man wanting to become a woman. Women who have accepted male domination tend to agree with that view” (Vidya, 2013, p. 122). Here, Vidya chooses to assert her self-constructed identity even when her family members berated her and her dad regularly thrashed her for resisting the imposed identities.

#### 3.2.3 Intense positive feelings and emotions

Trans women experience positive emotions by experimenting with feminine gender expressions and controlling external perceptions of bodies while feeling contentment in the expression of identified gender (Kulesza et al., 2025).

“Today, I wore a white sari with blue flowers printed on it that Gautami Amma gave me and helped me put it on. I placed a dot on my forehead, tied my hair in a bun and stood before the mirror. I was a woman —a beautiful woman” (Vidya, 2013, p. 62).

Vidya's embodiment of femininity in her sociocultural behavior reflects her psychological sense of self as a woman. She sculpts her expression and behaviors to be congruent with her identified gender identity. She feels alive only when she perceives her feminine image. She has to constantly defy the societal gender roles assigned to a man and adopt those associated with a woman to present herself as a woman.

### 3.3 Socially constructed identity

Discrepancies between an individual's innate gender identity and the socially assigned meanings of gender can lead to gender dysphoria (Paz Galupo et al., 2021a; Puvaneyshwaran et al., 2025). (Bourdieu 2010) theorizes that the disparity between the socially accepted body and the lived body is continuously conspicuous to outsiders and internalized by trans individuals (Perger, 2024). Consequently, to avoid the othering process and isolating experiences, a trans woman expresses herself through performative practices when interacting with heteronormative society, which is aligned with the “essentialised notions of cisfemininity” (Nair and Francis, 2024). Vidya adopts the social roles performed by other cis women in India by observing the women in her family, her circle of friends, and the cinema. This process leads to the satisfaction of euphoric factors, including a stimulus or gender-affirming antecedent, affirmation and validation, and intense positive emotions.

#### 3.3.1 Stimulus or gender-affirming antecedent

The social expression of gender identity is enacted through appearance, behavior, and conformity to social expectations (Ogle et al., 2023; Pardo, 2019), as it provides a deeper understanding of the perceived social self (Klussman et al., 2022; Wilson et al., 2024).

“As the days progressed, I started wishing Ilango would feast on me the way he enjoyed watching other girls. I became eager to win his love... Ilango was the man who kindled in me the kind of changes that occur in a woman at different stages of her development. Ilango was the man who made me feel whole as a woman” (Vidya, 2013, p. 28).

The patriarchal, heteronormative attraction prevalent in society influences Vidya's desire to be validated as a woman, particularly through securing the approving gaze of men. Films and everyday interactions with men around her stimulate corresponding ways of affirming her gender identity.

#### 3.3.2 Affirmation and validation

The identity validation of trans people occurs when assimilation happens in a social environment and when recognized with the desired gender identity (Tebbe et al., 2024).

“At the NGO, I regularly met people like me who went around in male garb but were women in spirit and urges—known as kothis. There were also those who openly wore women's clothes and looked feminine, some of whom had even undergone the sex change operation. These were known as tirunangais” (Vidya, 2013, p. 44).

Coming into contact with other trans people who share similar feelings, emotions, and experiences is highly affirming for Vidya. She perceives her existence as validated when she encounters trans women like her. When the peers from the trans community accept her, she feels socially relevant and accepted.

“The editor of Aval Vikatan, who had heard about my employment and stay in a hostel, contacted me with a view to featuring me in the magazine. I was not overly enthused by magazine articles on me, but I was happy that a women's magazine was interested in me as a woman. It was an acknowledgment of my womanhood” (Vidya, 2013, p. 116).

Vidya feels euphoric when a mainstream women's magazine approaches her to feature her in an article. The efforts of constructing her social identity pay off when she receives social recognition for her womanhood. It is the greatest acknowledgment of her transition by the outside world, as it fosters a strong sense of affirmation and empowers her self-expression, as emphasized by (Kulesza et al. 2025).

#### 3.3.3 Intense positive feelings and emotions

Trans people often face misgendering, which can lead to dysphoric feelings (Todd, 2024). To address this problem, small but meaningful actions by cisgender individuals—such as using a person's chosen name instead of dead name, using the pronouns one identifies with, and employing dignified language in descriptions—can generate intense positive emotions.

“My classmates Ramalakshmi and Subhasri treated me like a girl, making me feel at home. They used the intimate form of address among girls, ‘dee', freely with me. ‘Vidya, this pair of jeans looks good on you,' one of them said. Another advised me on the size of bindi I should affix on my forehead. A third recommended the use of turmeric for a better complexion. I spent a happy day in the company of girls” (Vidya, 2013, p. 103).

The above excerpt shows how even the smallest acknowledgment of her socially constructed self—such as the use of female pronouns by people who are not part of the community—can greatly induce euphoric feelings and play a significant role in affirming her identity.

### 3.4 Creatively constructed identity

Creatively constructed identity refers to the process of creatively accommodating one's identified gender while actively resisting the experiences of the assigned gender. Artistic manifestation of identities enhances “self-esteem, self-worth, social support, and social connections” (Austin et al., 2022a, p. 6). For instance, applied theater has been shown to function as a reflective and empowering tool in addressing LGBTQ-based aggression (Puvaneyshwaran et al., 2025). (Austin et al. 2022a) identify several themes of identity affirmation through creative expression, including “art as a: (1) form of authentic self-expression, (2) coping mechanism, (3) way to connect to others, and (4) pathway toward agency” (p. 16). These themes can be connected to Vidya's attainment of euphoric factors, namely a stimulus or gender-affirming antecedent, affirmation and validation, intense positive emotions, and, more importantly, enhanced quality of life through the creative process.

#### 3.4.1 Stimulus or gender-affirming antecedent

Art can be used as a guide to understand the transitioned self in the deepest sense and express the inner sense of identity despite experiencing opposition (Esterline, 2021).

“A river ran close to our home at Somarasampettai, in which I regularly bathed and washed clothes at seven in the morning... The isolation gave me the freedom to indulge my fancies; it awakened my feminine sensibilities enough to make me want to dance. In my imagination, I danced every time I bathed or washed clothes on those steps. Wrapping my towel around me, I swam in the canal and visualized a hero grabbing me in the water” (Vidya, 2013, p. 27).

The act of imagination provides Vidya with a sense of freedom to express her innermost emotions through song and dance within the confines of isolated spaces. Vidya also emulates the nuanced feminine expressions and behaviors of Tamil film stars such as “Meena, Roja, Nagma, and Rambha” and internalizes them (Vidya, 2013, p. 27).

“I masqueraded as the heroines, dressing and walking around like them. In this, I was aided and abetted by my sister Manju's skirts and midis, her eyeshadow, bangles, bindis and costume jewels. Lipstick was easily replicated by applying coconut oil to my lips and rubbing it in repeatedly. Long, plaited hair was an altogether different issue, but I knew how to overcome that problem too: just spread a thin cotton towel—a large kerchief, really (the kind that is common among ordinary folk, and not like Turkish towels)—on your head like a veil, not covering the forehead, and twirl the long rear portion as if it were covering a pigtail. Worn this way, you could easily pass as a girl drying her hair after a shower” (Vidya, 2013, p. 21).

The above excerpt aligns with Tanupriya and Pannikot's (2021) statement that trans women wish to grow long hair, dress exaggeratedly, and adopt feminine body language. The practice of secretly masquerading as film heroines significantly enables her to explore her desired sense of identity and helps her in gaining the experiences of being a woman from her childhood, even before her transition.

#### 3.4.2 Affirmation and validation

(Quach and Kwon 2025) emphasize that creative pursuits enable trans individuals to derive elation and recognition from others through the expression of self-affirmed identities, while also fostering feelings of comfort and contentment.

“My early posts on the blog were introductory pieces about me. Later, to my own surprise and pleasure, I wrote a couple of verses. I then joined a group called Tamizhmanam. My blog friends grew in number and variety quite rapidly: Divakar, Pons, Perasiriar, Dharumi, Ram, Mutturaman, Luckylook, Azhiyuran, Yogan Paris, and Sentazhal Ravi were some of my blogmates. They all encouraged me to continue writing... I poured out all I knew in my blog, based on my own experience. My writing had an impact on many people, with far-reaching effects” (Vidya, 2013, p. 115, 116).

Creative writing provides Vidya with a safe space to boldly put forward her personal narratives of identity and existence without concealment, even as she faces immense struggles in a transphobic society. It is evident that she gains validation and establishes a healthy social circle for herself by expressing her interests in books, poetry, blogging, and storytelling, which, in turn, affirms her sense of self-worth and belonging.

#### 3.4.3 Intense positive feelings and emotions

Gender euphoria can be achieved through self-expression and social connections (Tebbe et al., 2024). However, in contexts where the real world poses threats to individuals who openly express their identities, art can be used as a vital avenue for unveiling the true self and establishing real connections with people.

“On one occasion I acted like a girl for the benefit of Maharasan and a couple of other university students. Actually, I was pretending to imitate a girl for fun, and they liked my ‘acting'—but deep inside I was not really acting; I was subtly expressing my inner urges” (Vidya, 2013, p. 50).

From childhood, Vidya has yearned to share the gendered experiences of the women around her, including her sisters, aunt, mother, and female friends. When she expresses her femininity through a play, she experiences acceptance. Moreover, her artistic identity helps her build bonds with genuine allies, as reflected when her friend Bhupati says, “You are an artist, an angel. You should be our energy”. “The dam burst and tears flowed down my cheeks at this demonstration of love and affection by my friends, which came as a balm to the emotional wounds I had accrued” (Vidya, 2013, p. 108).

#### 3.4.4 Enhanced quality of life

Physically, mentally, and socially liberating experiences are gained through creative manifestation of identities. Art can be a source of self-affirmation through physically embodied performances, a coping mechanism for alleviating anxiety and depression (Kozee et al., 2012; Goetz and Arcomano, 2023; Ussher et al., 2023), and a means of achieving employment and social recognition (Austin et al., 2022a).

“I retreated to a world of my own creation. My sessions of dancing to the radio in drag continued, but extremely carefully—with great secrecy.” – it can be an avenue for self-soothing, assertive exploring by oneself” (Vidya, 2013, p. 25).

Vidya liberates her physicality through creative affirmation of her identified gender. She carries herself gracefully, internalizing, and embodying media representations of femininity. Sometimes, her family members and neighbors treat her badly for her involvement in art. Therefore, secrecy is the key factor in maintaining peace in Vidya's life until she gets the security and safety of being independent as a woman.

“My secret life was the best medicine for my depression, but even that was fraught with risk” (Vidya, 2013, p. 32).

Vidya experiences psychological freedom when she sings and dances. She says that, as a trans woman, susceptibility to harm and insults is inevitable, but art becomes a coping mechanism alongside being a tool for self-expression. In addition, through her writing, she transforms her harsh realities into cathartic narratives in her blogs.

“Balabharati suggested I start blogging. Soon, my blog was in place: http://livingsmile.blogspot.com... Before the article, I had only two friends in the hostel—Malar and her roommate. Now many readers, affected by the story, were viewing me like a celebrity.... office work, field work, conversations with my friends during leisure hours, friendship with my hostel mates and finally my active internet life— all made me happy and fulfilled” (Vidya, 2013, p. 115).

Art also liberates Vidya socially as it helps her gain support and connect with a large group of people. Through the spaces of creative expression, she expresses herself, and fellow theater members, understanding her plight, come forward to help her. She is also approached by a magazine team after they read her blogs.

The whole team of my well-wishers were on a job hunt for me—Nehru, Mu Ra, Murugabhupati, Selvam, Viji, all of them... My theatre friends were a group of happy people, who were together wherever they went... They embraced me with great compassion. (Vidya, 2013, p. 104, 108).

Here, art provides social recognition and opportunities, while partially enabling the financial agency needed to become the self-one aspires to be, including access to gender-affirming care and the creation of a sustainable life (Austin et al., 2022a). Due to the prevalence of social stigma, the trans community lives on the fringes of society (Wong et al., 2022), and transgender-inclusive spaces remain limited (Sharma, 2025). In such a scenario, Vidya uses art as a tool for activism.

Does anyone have the minimum awareness about us? We are objects of ridicule; film songs treat us as freaks. Every time I come across such lewdness, my blood boils. Why can't people who depict us so understand our pain and suffering? Society marginalizes us constantly. Tirunangais have no family, no jobs, no security, nothing... All I want is legal approval and recognition that will enable us to walk freely in public. Why can't governments think on these lines?... I do not ask for heaven—I am begging to be spared from living hell. I plead for myself and fellow tirunangais (Vidya, 2013, p. 121, 122).

In the final chapter of her autobiography, she appeals for recognition of her struggles and urges the government to think about the welfare of minority trans communities. Through her meaningful art, Vidya not only enriches her own life but also serves as a representative, positively impacting the lives of other trans individuals. Trans people engaged in creative expressions of lives liberate everyone and become active agents of change (Puvaneyshwaran et al., 2025). Art provides trans people with recognition, acceptance, and agency to raise awareness, foster empathy, advocate for rights, and amplify voices for the upliftment of the whole trans community. Therefore, visibility works through art in three ways: “visibility of the self, seeking genuine recognition or visibility from others, and aspiring to establish platforms of visibility for individuals with similar marginalized identities” (Parker, 2024).

Table 1 illustrates that a stimulus or gender-affirming antecedent, affirmation and validation, and positive feelings and emotions responsible for gender euphoria are attained through physical embodiment, self-constructed identities, and socially constructed identities. Creative construction provides an additional euphoric dimension in the form of enhanced quality of life, thereby satisfying all the conditions for gender euphoria outlined in the research of (Austin et al. 2022b) and (Leitch et al. 2025). Previous research has primarily linked gender euphoria to preexisting trans-identity formations within biological, psychological, and social constructivist frameworks. The present analysis substantiates that identity construction through creative expression, in combination with the previously identified aspects, contributes to all the factors necessary for the attainment of gender euphoria. Trans people can experience euphoria to the fullest, as they feel confident, uninhibited, appreciated, and resilient when immersed in artistic expression, which produces significant long-term benefits associated with gender euphoria.

**Table 1:** Analysis of identity construction and gender euphoria attainment in Living Smile Vidya's autobiography, *I Am Vidya: A Transgender's Journey* (2013).

**Aspects of identity**	**Themes identified from *I Am Vidya***	**Gender-euphoric factors attained by Vidya**
Physical embodiment	Desperation for internal alignment	Stimulus or gender-affirming antecedent
	Contentment with one's body	Intense positive feelings and emotions
Self-constructed identity	Influence of gender stereotypes	Stimulus or gender-affirming antecedent
	Adopting gender stereotypes	Affirmation and validation
	Embodying the sociocultural behavior of the identified gender	Intense positive feelings and emotions
Socially constructed identity	Gender-specific expectations of the identified gender	Stimulus or gender-affirming antecedent
	Assimilation with fellow people	Affirmation and validation
	Receiving acknowledgment	Intense positive feelings and emotions
Creatively constructed identity	Art as a stimulus for authentic self-expression	Stimulus or gender-affirming antecedent
	Widened social connections	Affirmation and validation
	Avenue for expression, appreciation, and acknowledgment	Intense positive feelings and emotions
	Art as a coping mechanism, liberating experience, and pathway toward agency	Enhanced quality of life

## 4 Discussion

Analysis of Living Smile Vidya's autobiography through the Extended Theory of Trans-Identity reveals that Vidya's ongoing quest for identity validation reaches full fruition when she starts expressing herself through creative avenues such as dance, theater, and writing. She physically embodies femininity through her bodily movements, dialogue, and expressions in creative arts; asserts herself by voicing her inner thoughts and emotions both privately and publicly; and gains social recognition as her identity is communicated through art. Thus, the manifestation of the creative self, in combination with the physical, self-constructed, and socially constructed aspects of identity, contributes to the attainment of gender euphoria. The results of the analysis indicate that motivating a trans person to engage in any form of art can foster personal agency and facilitate acceptance of a redefined self. In this discriminating world, trans people must adopt multiple strategies to affirm desired gender identities. Art not only helps one achieve a sense of belonging but also lays the foundation for an optimistic future, highlighting the importance of trans inclusivity.

The interplay of creative identity construction with the other three aspects of identity is further illustrated by the success stories of eminent trans women in India, namely A. Revathi (writer and theater artist), Kalki Subramaniam (writer and actor), and Nega Shahin (actor). A. Revathi, a Bharatanatyam dancer, theater artist, and writer, who has achieved remarkable success in affirming her identity as a woman, undergoes a physical transition, constructs herself independently, and socially identifies as a woman. In addition to these efforts, she turns to writing, shaping her lived experiences into an autobiography titled *The Truth About Me: A Hijra Life Story* (2010) to create awareness and advocate for the inclusivity of trans people. Despite being rejected even after undergoing a physical transition and making every effort to assert her true identity, she has been acknowledged for her writing and is recognized as one of the greatest women writers of the world, who has been honoured on the walls of Columbia University. Evidence of this historical moment is documented in P Abhijit's *I am Revathi* (2025), a documentary film on her. Kalki Subramaniam, another trans woman, calls herself an “artivist,” having recognized the power of creative expression to drive activism against trans discrimination and to provide tangible support to trans communities. Nega Shahin, a trans woman, has received the Kerala State Award for Acting for her role in the film *Antharam* (2022), which explores trans experiences.

## 5 Conclusion

The article highlights the positive significance of being involved in art, showing how transgender individuals can affirm identities in a better way through the dynamic integration of physically, psychologically, socially and creatively constructed aspects of the self. The expression of authentic gender identity through the artistic medium is a reinforcing process, as it facilitates recognition from others and generates gender-euphoric feelings as a reward for self-expression. The trans realities presented in this article serve as evidence for developing an extended version of the trans-identity framework, emphasizing the central role of creativity in the gender-affirmative journeys of trans people.

## 6 Limitations

First, the extended version substantiates the theory through the experiences of trans women, with no inclusion of trans men's perspectives. Second, the researcher lacks first-hand experience of the challenges faced by trans people and relies solely on a selected autobiographical text of a trans woman from India to explore the lived realities of Indian trans women. Third, the creative aspect of identity formation, as delineated in the paper, proves particularly relevant for trans women who engage in or are interested in creative practices and may not be universally applicable to all trans women.

## Data Availability

The original contributions presented in the study are included in the article/supplementary material, further inquiries can be directed to the corresponding author.
